# Kutane Läsion in der Axilla – eine (außer)gewöhnliche Diagnose

**DOI:** 10.1007/s00292-026-01556-9

**Published:** 2026-04-13

**Authors:** Sarah Theurer, Sabrina Borchert, Elisabeth Bednaric, Hideo A. Baba, Lutz Schmitz

**Affiliations:** 1 Institut für Pathologie, Universitätsklinikum Essen, Universität Duisburg-Essen, Hufelandstr. 55, 45147 Essen, Deutschland; 2 Dermatohistopathologie Centroderm, Wuppertal, Deutschland

**Keywords:** Granularzelltumor, Kutaner nichtneuronaler Granularzelltumor, Primitiver polypoider Granularzelltumor, ALK-Fusion, Hauttumor, Granular cell tumor, Cutaneous non-neuronal granular cell tumor, Primitive polypoid granular cell tumor, ALK fusion, Skin tumor

## Abstract

Bei einer 21-jährigen Patientin wurde ein 1 cm großer, rot-polypoider Hauttumor nahe der Axilla exzidiert. Histomorphologisch zeigte sich ein subepithelialer Tumor mit Charakteristika eines Granularzelltumors, jedoch waren S100- und SOX10-Immunhistochemie negativ. Eine zusätzliche *ALK*-Immunhistochemie war positiv und mittels Archer-Analyse wurde eine *KLC1*–*ALK*-Fusion (KLC1 Exon 3–ALK Exon 20) nachgewiesen. Auf dieser Grundlage wurde die Diagnose eines kutanen nichtneuronalen Granularzelltumors gestellt. Dabei handelt es sich um eine seltene kutane Neoplasie mit histomorphologischer Ähnlichkeit zu klassischen Granularzelltumoren, jedoch ohne deren typisches immunhistochemisches Profil. In den meisten Fällen liegt eine *ALK*-Fusion zugrunde, die auch immunhistochemisch durch *ALK*-Expression nachgewiesen werden kann.

## Abstract (en)

A 21-year-old female presented with a 1 cm red, polypoid cutaneous tumor near the axilla that was surgically excised. Histomorphology revealed a subepithelial tumor with features suggestive of a granular cell tumor; however, immunohistochemistry for S100 and SOX10 was negative. Further analysis showed immunohistochemical *ALK* positivity, and Archer-based molecular testing identified a *KLC1*–*ALK* fusion (KLC1 exon 3–ALK exon 20). These findings supported the diagnosis of a cutaneous non-neural granular cell tumor, a rare cutaneous neoplasm that mimics conventional granular cell tumors histomorphologically but lacks their typical immunophenotype and commonly harbors *ALK* fusions.

## Anamnese/klinische Angaben/Makroskopie

**Abb. 1:**
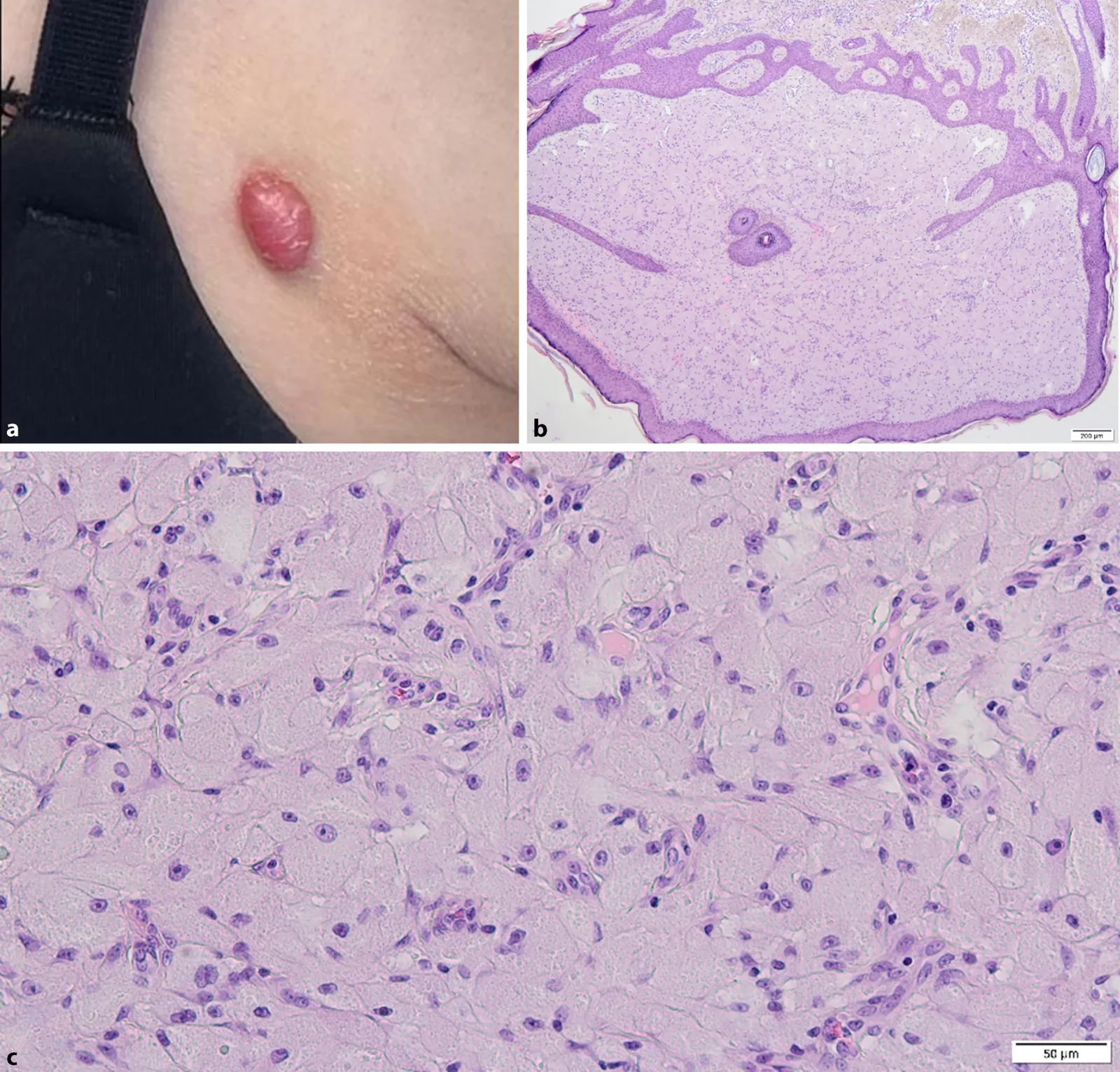
Makroskopisch imponierte nahe der linken Axilla ein erhabener, polypoider Tumor von rötlicher Farbe (**a**). HE-morphologisch stellte sich ein subepithelial gelegener Tumor dar, der die Haut polypoid vorwölbte (**b**, HE, Vergr. 40:1). Die Zellleiber voluminös mit einem blass eosinophilen, feingranulären Zytoplasma (**c**, HE, Vergr. 200:1)

Bei dem dargestellten Fall wurde ein Hautexzidat aus der linken Axilla einer 21 Jahre alten weiblichen Patientin eingesandt. Die klinische Fragestellung lautete „Narbenkeloid?“. Über eine Voroperation in diesem Gebiet ist nichts bekannt. Makroskopisch imponierte ein polypoid erhabener, rötlicher Tumor nahe der Achselfalte von ca. 1 cm Größe und 0,7 cm Erhabenheit (Abb. 1a).

## Histomorphologie

**Abb. 2:**
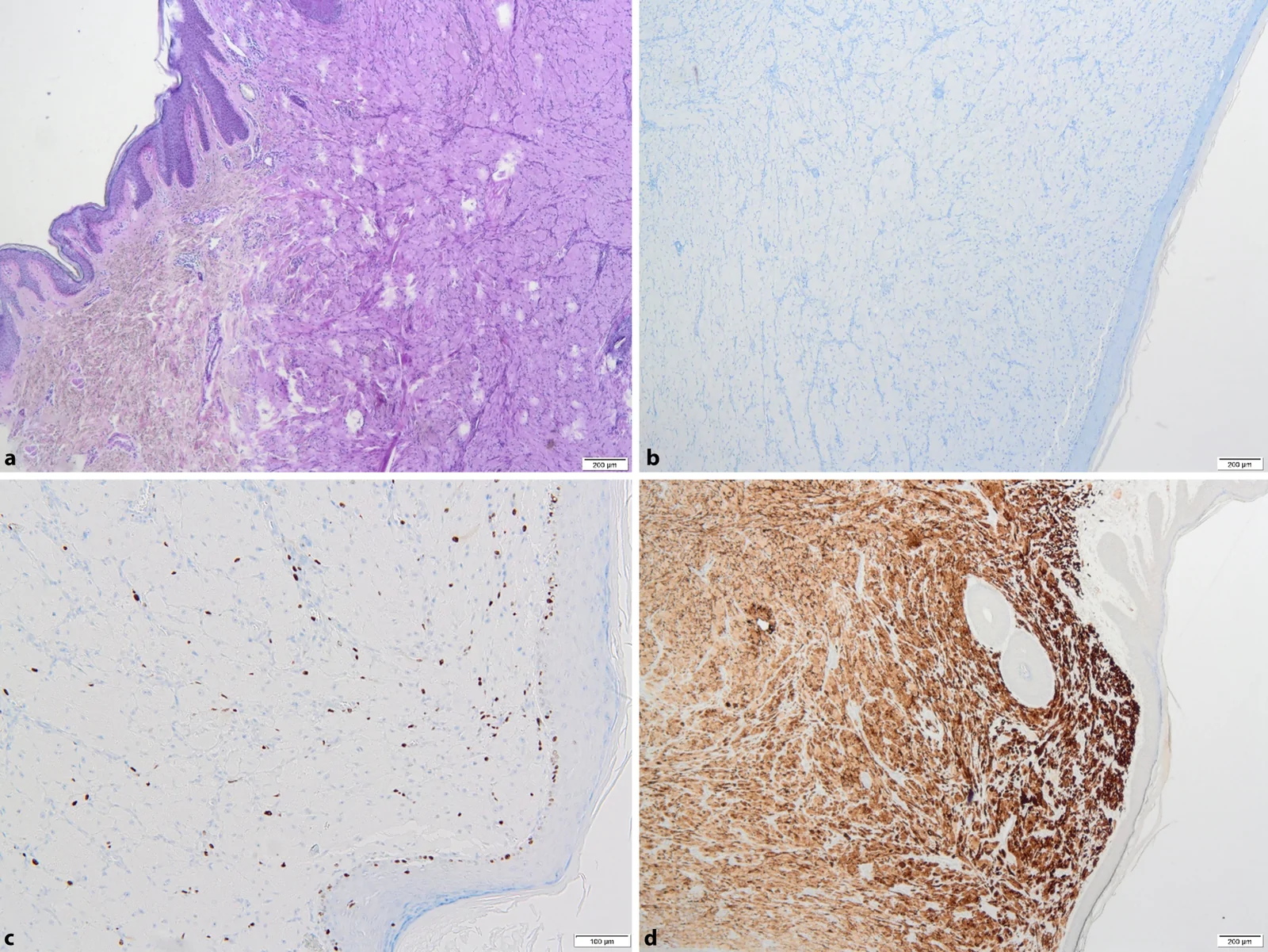
Ergebnisse der Spezialfärbungen. In der PAS-Färbung waren die Tumorzellen positiv (**a**, PAS, Vergr. 40;1). Die immunhistochemische Reaktion für S100 blieb negativ (**b**, S100, Vergr. 40:1) im Gegensatz zum Färbeverhalten klassischer Granularzelltumoren. Typischerweise ist die proliferative Aktivität mit Ki67 niedrig (**c**, Ki67, Vergr. 100:1). Der Nachweis der Entität gelang über die immunhistochemische Färbung mit ALK (**d**, ALK D5F3, Vergr. 40:1)

Histopathologisch zeigte sich in der HE-Färbung ein Hautexzidat mit einem unmittelbar subepithelial gelegenen Tumor, der die Haut polypoid vorwölbte und die Hautadnexen aussparte (Abb. 1b). Das den Tumor überkleidende Epithel ohne (reaktive) Hyperplasie. Der Tumor selbst bestand aus großleibigen Zellen, die in dichten, soliden Verbänden mit beinahe synzytialem Aspekt lagen. Die Zellkerne waren mittelgroß und wenig pleomorph, einige zeigten Makronukleolen. Das Zytoplasma war feingranulär (Abb. 2c). Bei der PAS-Reaktion zeigte sich eine positive Reaktion im Vergleich zur Umgebungsreaktion (Abb. 2a).

**Verdachtsdiagnose:** Granularzelltumor

## Weiterer Verlauf

Es wurden immunhistochemische Untersuchungen angeschlossen, um die Verdachtsdiagnose zu bestätigen. Überraschenderweise waren die typischen Marker des Granularzelltumors S100 und SOX10 negativ (Abb. 2b). Die CD68-Färbung zeigte ein schwach positives Ergebnis. Die proliferative Aktivität war mit ca. 5 % Ki67-positiver Zellkern sehr niedrig (Abb. 2c). Es wurde eine ALK-Färbung (Klon D5F3) angeschlossen, die kräftig positiv ausfiel (Abb. 2d). Die zur Bestätigung der differentialdiagnostischen Überlegungen angeschlossene Archer-Untersuchung bestätigte eine *ALK*-Fusion, hier mit dem Partner *KLC1* (KLC1 [NM_005552.4] Exon 3 – ALK [NM_004304.5] Exon 20).

**Diagnose:** kutaner, nichtneuronaler Granularzelltumor (primitiver, polypoider Granularzelltumor)

## Diskussion

Kutane nichtneuronale Granularzelltumoren (NNGCT) sind äußerst seltene Neoplasien, die bislang nur in wenigen Fallberichten und kleinen Fallserien beschrieben sind und erstmals 1991 von LeBoit dokumentiert wurden [1]. Klinisch imponieren sie als polypoide, rötliche Tumoren, die in allen Altersgruppen bei beiden Geschlechtern auftreten können. Sie können am kompletten Integument entstehen und zeigen in der Regel ein langsames Wachstum, sodass sie oft bereits mehrere Monate vor Exzision bestehen [2].

Histomorphologisch ähneln NNGCT klassischen Granularzelltumoren. Charakteristisch sind große, polygonale oder spindelige Tumorzellen mit voluminösen, onkozytären Zellleibern und feingranulärem Zytoplasma. Die Zellkerne sind typischerweise rund mit kleinen Nukleolen [1–3]. Zytologische Atypien und eine erhöhte mitotische Aktivität können vorkommen. Die überkleidende Epidermis kann, muss aber nicht, eine reaktive Hyperplasie aufweisen [4]. Im Gegensatz zu typischen Granularzelltumoren zeigen NNGCT keine immunhistochemische Expression von S100 und SOX10, exprimieren jedoch unspezifisch CD68 und CD10. Die Mehrzahl der Fälle ist immunhistochemisch ALK-positiv, was mit dem Nachweis einer zugrunde liegenden *ALK*-Fusion korreliert. In den wenigen bislang dazu publizierten Fällen wurden *DCTN1* und *SQSTM1* als Fusionspartner identifiziert [4]. Es existieren jedoch auch Berichte über Fälle ohne nachweisbare ALK-Expression und/oder *ALK*-Fusion. In diesen meist älteren Fällen wurde die Diagnose nach histomorphologischen Kriterien und aufgrund der fehlenden immunhistochemischen Expression der für Granularzelltumoren typischen Marker gestellt [4]. Die in unserem Fall nachgewiesene *ALK-KLC1*-Fusion ist bislang für NNGCT nicht beschrieben, jedoch als pathogene Alteration in Lungenkarzinomen bekannt [5].

Nach vollständiger Exzision gelten die Patienten in den meisten Fällen als kuriert. Nur vereinzelt sind Verläufe mit Ausbildung von Metastasen dokumentiert [6, 7]. Diese Fälle wurden jedoch nicht hinsichtlich einer ALK-Expression oder *ALK*-Fusion untersucht, sodass offenbleibt, ob es sich bei diesem, offenbar aggressiver verlaufenden Subtyp möglicherweise um eine eigenständige Tumorentität handelt.

## Fazit für die Praxis

- Auch bei typischer Histomorphologie sollte ein Granularzelltumor immunhistochemisch bestätigt werden, um die (seltene) Differentialdiagnose eines nichtneuronalen Granularzelltumors zu berücksichtigen.
- Eine Bestätigung dieser seltenen Diagnose gelingt über die ALK-Färbung oder den Nachweis einer *ALK*-Fusion, die in der Mehrzahl der Fälle zu finden ist.

## Data Availability

Die Daten zu diesem Artikel sind auf Anfrage bei den Autoren verfügbar.
